# Promoter polymorphisms in the lncRNA-MIAT gene associated with acute myocardial infarction in Chinese Han population: a case–control study

**DOI:** 10.1042/BSR20191203

**Published:** 2020-02-24

**Authors:** Ruchao Ma, Xiaohui He, Xiaoyun Zhu, Shuchao Pang, Bo Yan

**Affiliations:** 1 Shandong University Cheeloo College of Medicine, Jinan, Shandong 250012, China; 2 Department of Cardiology, Lanzhou University Second Hospital, Lanzhou, Gansu 730030, China; 3 Division of Gastroenterology and Hepatology, Gansu Provincial Hospital, Lanzhou, Gansu 730030, China; 4 The Center for Molecular Genetics of Cardiovascular Disease, Affiliated Hospital of Jining Medical University, Jining Medical University, Jining, Shandong 272029, China; 5 Shandong Provincial Key Laboratory of Cardiac Disease Diagnosis and Treatment, Affiliated Hospital of Jining Medical University, Jining Medical University, Jining, Shandong 272029, China

**Keywords:** Acute myocardial infarction, Coronary atherosclerotic disease, Myocardial infarction associated transcript, Polymorphism

## Abstract

**Background:** Coronary atherosclerotic disease (CAD) is one of the greatest causes of death and disability around the world, and has emerged as a major public health problem. Acute myocardial infarction (AMI) is the most serious type of CAD. Myocardial infarction (MI) association transcript (MIAT) has demonstrated that it plays an important role in AMI. **Purpose:** To investigate the association between MIAT promoter polymorphisms and AMI in Chinese Han population. **Methods:** A total of 212 AMI patients and 218 healthy controls were recruited. The long non-coding RNA (lncRNA)-MIAT promoter polymorphisms (single nucleotide polymorphisms (SNPs)) were obtained using polymerase chain reaction (PCR) and sequencing techniques. Chi-square test was used to analyze the allele and genotype frequencies of each SNP in two groups. Logistic regression analysis was used to analyze the association of each SNP with AMI. Linkage disequilibrium (LD) and haplotype analysis were performed using SHEsis software. A JASPAR database search predicts transcription factors transition of linked polymorphism in MIAT promoter. **Results:** Ten SNPs were found, including rs56371714, rs55892869, rs151057042, rs2157598, rs150465374, rs5761664, rs8142890, rs5752375, rs9608515 and rs1055293700, whereas rs1055293700 was found only in the control group. Single and logistic regression analysis showed that there was a significant correlation between rs5752375 and rs9608515 polymorphisms and AMI, while other sites had no relationship with AMI. These MI association polymorphisms may change the binding sites with transcription factor. **Conclusions:** The polymorphisms of lncRNA-MIAT promoter rs5752375 and rs9608515 were significantly associated with AMI in Chinese Han population. This result would be of clinical importance for the early diagnosis of AMI.

## Introduction

Coronary atherosclerotic disease (CAD) is one of the greatest causes of death and disability around the world [1], and has emerged as a major public health problem [2]. To date, the etiologies of CAD have not been completely explained. Epidemiological studies have suggested that traditional risk factors such as age, sex, smoking, hypertension, diabetes may play crucial roles in the CAD development and pathologies [3]. In addition to these factors, many researches found that genetic factors participate in regulation of physiological or pathologies in life [4–6], and have reported many genetic factors link with CAD. At the beginning of exploration in genetic factors, the coding genes were focused on and found in CAD, such as *APOM* gene [7], *APOE* gene [8] and low-density lipoprotein (LDL) receptor-related protein 6 gene [9]. However, the beginning of study of gene mainly focused on protein-coding genes, the majority of the genome was defined non-coding DNA or ‘junk’ DNA. With the completion of human genome project and approved that the number of protein-coding genes is very small, which is less than 2% of entire genome [10]. With the development of molecular biology and accompanied with high-throughput sequencing appearance, the direction of gene research turn to no-coding gene, the sequence and function of partial non-coding genes were classification and annotation [11], which suggested that non-coding RNA linked with many diseases [12].

Long non-coding RNA (lncRNA) is a member of the RNA family, with length of more than 200 nucleotides. In recent years, numerous studies have approved lncRNA involved in biological processes including proliferation, differentiation and apoptosis [13–15]. X-inactive specific transcript (XIST) is a dramatic example of lncRNA, which plays a key role in silencing of X-chromosome [16]. LncRNA also takes part in processes of many diseases including CAD [6], such as NEXN-AS1 which was indicated as potential therapeutic target in CAD [17]. Myocardial infarction (MI) as one of the most serious types for CAD, a plenty of lncRNAs were noted and linked with MI [18]. MI association transcript (*MIAT*) gene encodes a spliced lncRNA that may constitute a component of the nuclear matrix association, which is located at the long arm of chromosome 22 (22q12.1) and five exons were transcripted by it [19,20]. Since it was discovered by researches in 2006, it was considered as a risk for MI [21]. In recent years, growing evidence has indicated that MIAT plays an important role in schizophrenia [22,23], diabetic retinopathy [24], neuroendocrine prostate cancer [25], cardiac hypertrophy [26] and acute MI (AMI) [27]. Currently, a related research has demonstrated that the expression of MIAT in atherosclerotic plaque is higher [28], which results were verified in animal model [29]. In patients with AMI treated by primary percutaneous coronary intervention, MIAT is lower expression in ST-segment-elevation MI compared with non-ST-segment-elevation MI [27]. These studies showed that MIAT has positive correlation with AMI.

Previous researches have indicated that single nucleotide polymorphisms (SNPs) of coding gene take part in CAD [30], and results were verified by meta-analysis [31]. The SNPs of lncRNA also contribute to the risk of CAD, such as SNPs in *MALAT1* gene [32], in addition, the meta-analysis about SNPs of lncRNA in CAD were published [33]. Genome-wide association studies have identified some genetic variants of MIAT related to MI [21]. However, no research has explored the association between SNPs in MIAT promoter and AMI risk in a Chinese Han population.

## Methods

### Study populations

In the present study, 430 people were recruited including 212 AMI patients and 218 ethnic-matched healthy controls in Affiliated Hospital of Jining Medical University from September 2016 to March 2018. All AMI patients were diagnosed by clinical guide of AMI in Cardiac Care Unit, including clinical symptoms, electrocardiogram and the level of myocardial enzyme. The healthy controls did not have familial CAD history. All study populations had no history of other cardiovascular diseases, such as cardiomyopathy, congenital heart disease. All subjects provided written informed consent, and this research was designed according to the principles of the Declaration of Helsinki and was approved by ethics committee of Affiliated Hospital of Jining Medical University.

### DNA sequencing

The collection of blood samples were from peripheral venous and leukocytes were isolated, and genomic DNAs were extracted from leukocytes. The proximal promoter region of the lncRNA-MIAT gene (1294 bp, from −1063 to +231 bp) was analyzed. In order to improve the accurate result, the total sequence was divided into two overlapped fragments to amplify by polymerase chain reaction (PCR). PCR primers were designed based on genomic sequence of the human lncRNA-MIAT gene (NCBI, NG_016621.2) (Table 1). The amplification of PCR was done by Applied Biosystems 9700 PCR instrument, and PCR products were directly sequenced by Applied Biosystems 3500XL genetic analyzer in Shanghai Sangon Biotech. The results of sequence were analyzed by Chromas and DNAMAN software.

**Table 1:** The primers of two overlapped fragments

Fragment	PCR primers	Sequences	Length of primers	Products
Fragment 1	MIAT-F1	5′-GGTTCGAGTGATTCTAGTGCC-3′	21 bp	703 bp
	MIAT-R1	5′-CCCGGAGTAAGAGGACAGAA-3′	20 bp	
Fragment 2	MIAT-F2	5′-GACTCCACCATAACCATGTGCA-3′	22 bp	696 bp
	MIAT-R2	5′-CCGTTAGGGGACAAGGACACT-3′	21 bp	

### Statistical analysis

The values of quantitative data were presented in mean ± standard, and analyzed by Student’s *t* test. The qualitative data were analyzed by Chi-square test. The Hardy–Weinberg equilibrium test was used to analyze the frequency of allele distribution of control group. The association between SNPs and AMI was analyzed by using binary logistic regression. The risks were assessed by calculating odds ratio (OR) and 95% confidence interval (95% CI). The ORs were adjusted for age, sex, hypertension, smoking, diabetes and the odds of LDL with high-density lipoprotein (HDL). All the data were analyzed by SPSS v20.0. The SHEsis software was used to analyze linkage disequilibrium (LD) and haplotype analysis. The statistical significance was considered as *P*<0.05.

### Prediction of transcription factor for polymorphisms

The website JASPAR (http://jaspar.genereg.net/) [34] was used to predict associated transcription factors of polymorphisms in promoter of lncRNA-MIAT. All transcription factors of *Homo sapiens* were selected to match with sequence of polymorphisms, the length of linked sequence was set at 15 bases before and after the site of polymorphism. Sensitivity and specificity were set in default 80%.

## Results

### Clinical characteristics

The present study recruited 212 AMI patients and 218 controls. The mean age of controls was 44.43 ± 12.23 years, and the group of patients included 138 males (63.30%) and 80 females (36.70%). The mean age of patients was 60.92 ± 12.26 years, and this group included 161 males (75.94%) and 61 females (24.06%). Demographic and clinical characteristics are collected in Table 2. Patients with AMI had significantly higher prevalence of traditional risk factors such as history of smoking (*P*<0.001), hypertension (*P*<0.001) and diabetes (*P*<0.001) than controls. In blood biochemical index, there was a significant difference in two groups, the total cholesterol (TC), triglycerides (TGs) and LDL is higher in case group compared with controls, however, the HDL is lower in case group, and statistical analysis indicated that the difference was significant in TC (*P*=0.05), TG (*P*<0.01) and HDL (*P*<0.01) between two groups.

**Table 2:** Characteristics of the study population

	Controls (*n*=218)	Cases (*n*=212)	*P*-value
Age (years, mean ± SD)	44.43 ± 12.23	60.92 ± 12.26	<0.01
Gender (%)			<0.01
Male	138 (63.30%)	161 (75.94%)	
Female	80 (36.70%)	61 (24.06%)	
BMI (kg/m^2^)	25.16 ± 3.47	25.72 ± 3.63	0.26
TG (mmol/l)	1.26 ± 0.84	1.45 ± 1.07	0.05
TC (mmol/l)	4.88 ± 1.67	4.33 ± 1.02	<0.01
HDL (mmol/l)	1.26 ± 0.31	1.14 ± 0.24	<0.01
LDL (mmol/l)	2.67 ± 0.78	2.76 ± 0.75	0.25
Smoking			<0.01
Yes	106 (17.43%)	38 (50.00%)	
No	112 (82.57%)	174 (50.00%)	
Diabetes			<0.01
Yes	48 (8.26%)	18 (22.64%)	
No	178 (91.74%)	194 (77.36%)	
Hypertension			<0.01
Yes	98 (26.61%)	58 (46.23%)	
No	120 (73.39%)	154 (53.77%)	

Abbreviations: case: AMI; BMI, body mass index; SD, standard deviation.

### Hardy–Weinberg equilibrium in controls

Ten SNPs were found in the control group, but since rs1055293700 appeared only in one sample, Hardy–Weinberg equilibrium test was performed in other nine SNPs. The result presented that all polymorphisms followed the Hardy–Weinberg equilibrium (*P*>0.05) except rs5761664, which indicated that the sample was derived from the genetic balance and was well represented. The results of Hardy–Weinberg equilibrium are shown in Table 3.

**Table 3:** Hardy–Weinberg equilibrium test in controls

Polymorphisms	Genotypes	Controls (*n*=218)		Hardy–Weinberg	
		Observation value	Expected value	X^2^ value	*P*-value
g.4004C>T(rs56371714)	CC	175	174.43	0.17	0.68
	CT	40	41.15		
	TT	3	2.43		
g.4063T>C(rs5752375)	TT	4	2.75	0.71	0.40
	TC	41	43.49		
	CC	173	171.75		
g.4112C>T(rs55892869)	CC	174	172.64	0.88	0.35
	CT	40	42.72		
	TT	4	2.64		
g.4137T>C(rs9608515)	TT	5	2.98	1.75	0.19
	TC	41	45.03		
	CC	172	169.98		
g.4359insG(rs151057042)	−/−	199	199.41	0.45	0.50
	C/−	19	18.17		
g.4445A>T(rs2157598)	AA	105	109.5	2.18	0.14
	AT	99	90.01		
	TT	14	18.50		
g.4453insA(rs150465374)	−/−	192	190.9	1.54	0.21
	A/−	24	26.20		
	AA	2	0.90		
g.4675C>T(rs5761664)	CC	97	103.90	4.78	
	CT	107	93.20		0.03
	TT	14	20.90		
g.4933T>C(rs8142890)	TT	5	2.98	1.75	0.19
	TC	41	45.03		
	CC	172	169.98		

### Allelic distribution and the frequency of genetic variants in two groups

**Figure 1:**
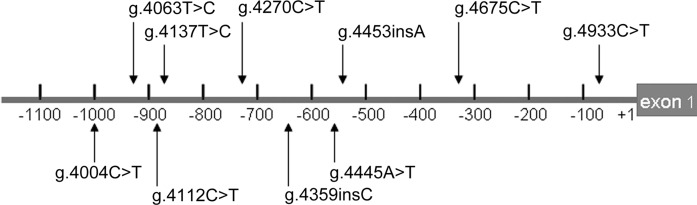
Locations and sequencing chromatograms of the SNPs in the lncRNA-MIAT gene promoter The numbers represents the genomic DNA sequences of the human lncRNA-MIAT gene (Genbank accession number NG_016621.2) upstream to the transcription start site (+1).

The allelic distribution and the frequency of genetic variants in two groups were presented in Table 3. In the present study, ten SNPs were found in two groups, which are g.4004C>T (rs56371714), g.4063T>C (rs5752375), g.4112C>T (rs55892869), g.4137T>C (rs9608515), g.4359insG (rs151057042), g.4360G>T (rs1055293700), g.4445A>T (rs2157598), g.4453insA (rs150465374), g.4675C>T (rs5761664), g.4933T>C (rs8142890), respectively. The location of the SNPs in the lncRNA-MIAT gene promoter showed in Figure 1. Statistical analysis was performed on all SNPs, but since the rs1055293700 only appeared in the control group, the other nine SNPs were statistically analyzed finally. We observed statistical difference in rs5752375 (*P*=0.02) and rs9608515 (*P*=0.04) polymorphisms between case group and control group by χ^2^ test, however, no statistical difference was found in rs56371714, rs55892869, rs151057042, rs2157598, rs150465374, rs5761664, rs8142890. The results of allelic distribution and the frequency of genetic variants in two groups are shown in Table 4.

**Table 4:** The polymorphisms in MIAT promoter between cases and controls

Polymorphisms	Genotypes	Location	Controls (*n*=218)	Cases (*n*=212)	*P*-value
g.4004C>T(rs56371714)	CC	−997	173	175	0.45
	CT		33	40	
	TT		6	3	
g.4063T>C(rs5752375)	TT	−938	15	4	0.02
	TC		31	41	
	CC		166	173	
g.4112C>T(rs55892869)	CC	−889	172	174	0.67
	CT		34	40	
	TT		6	4	
g.4137T>C(rs9608515)	TT	−864	15	5	0.04
	TC		31	41	
	CC		166	172	
g.4359insG(rs151057042)	−/−	−642	186	199	0.23
	C/−		26	19	
g.4360G>T(rs1055293700)	GT	−641	0	1	−
g.4445A>T(rs2157598)	AA	−556	115	105	0.26
	AT		80	99	
	TT		17	14	
g.4453insA(rs150465374)	−/−	−548	197	192	0.13
	A/−		15	24	
	AA		0	2	
g.4675C>T(rs5761664)	CC	−326	99	97	0.88
	CT		99	107	
	TT		14	14	
g.4933T>C(rs8142890)	TT	−68	2	5	0.52
	TC		38	41	
	CC		172	172	

The location of transcript start site was defined as +1.

### Association between polymorphisms and AMI

Logistic regression was performed on all polymorphisms to explore the association in the present study. The higher genotype of polymorphisms was defined as wild-type. The results showed there was a significant correlation between rs5752375 and rs9608515 and AMI, while other polymorphisms rs56371714, rs55892869, rs151057042, rs2157598, rs150465374, rs5761664 and rs8142890 had no correlation with AMI. For the rs5752375 polymorphism, the risk of TT genotype was 3.91-times higher than CC genotype AMI [OR = 3.91, 95% CI (1.27, 12.02), *P*=0.02]; for rs9608515 polymorphism, TT genotype was higher than CC genotype and the risk of AMI increased 3.11-times [OR = 3.11, 95% CI (1.10, 8.74), *P*=0.03]. After adjusting for risk factors such as gender, age, smoking, hypertension and diabetes, the ratio of LDL and HDL, the correlation between these polymorphisms and AMI was analyzed again. The difference was still statistically significant. For the rs5752375 polymorphism, the TT genotype was found to be a risk factor for AMI compared with the CC genotype [OR = 7.11, 95% CI (1.64, 30.79), *P*=01]; for the rs9608515 polymorphism, the TT genotype is still a risk factor for the AMI [OR = 5.56, 95% CI (1.43, 21.64), *P*=01]. The other polymorphisms rs56371714, rs55892869, rs151057042, rs2157598, rs150465374, rs5761664 and rs8142890 were still not linked with AMI. The results of association between polymorphisms and AMI are shown in Table 5.

**Table 5:** The results of association between polymorphisms and AMI

Polymorphisms	Genotypes	Non-adjusted		Adjust	
		OR (95% CI)	*P*-value	OR (95% CI)	*P*-value
g.4004C>T(rs56371714)	CC	1.0		1.0	
	CT	0.83 (0.50, 1.38)	0.48	0.63 (0.30, 1.29)	0.21
	TT	2.02 (0.50, 8.22)	0.32	2.51 (0.38, 16.64)	0.34
g.4063T>C(rs5752375)	CC	1.0		1.0	
	TC	0.79 (0.47, 1.32)	0.36	0.65 (0.32, 1.36)	0.26
	TT	3.91 (1.27, 12.02)	0.02	7.11 (1.64, 30.79)	0.01
g.4112C>T(rs55892869)	CC	1.0		1.0	
	CT	0.86 (0.52, 1.42)	0.56	0.73 (0.36, 1.48)	0.39
	TT	1.52 (0.42, 5.47)	0.52	2.24 (0.38, 13.40)	0.38
g.4137T>C(rs9608515)	CC	1.0		1.0	
	TC	0.78 (0.47, 1.31)	0.35	0.66 (0.32, 1.38)	0.27
	TT	3.11 (1.10, 8.74)	0.03	5.56 (1.43, 21.64)	0.01
g.4359insG(rs151057042)	−/−	1.0		1.0	
	C/−	1.46 (0.78, 2.73)	0.23	1.34 (0.55, 3.24)	0.52
g.4445A>T(rs2157598)	AA	1.0			
	AT	0.74 (0.50, 1.10)	0.13	0.62 (0.36, 1.07)	0.09
	TT	1.11 (0.52, 2.36)	0.79	1.24 (0.45, 3.41)	0.68
g.4453insA(rs150465374)	−/−	1.0			
	A/−	0.61 (0.31, 1.20)	0.15	1.02 (0.39, 2.69)	0.97
	AA	0.00 (0.00, Inf)	0.98	0.00 (0.00, Inf)	0.98
g.4675C>T(rs5761664)	CC	1.0		1.0	
	CT	0.91 (0.61, 1.34)	0.62	0.77 (0.45, 1.32)	0.35
	TT	0.98 (0.44, 2.16)	0.96	1.23 (0.43, 3.56)	0.70
	TT	1.0			
g.4933T>C(rs8142890)	TC	0.93 (0.57, 1.51)	0.76	0.82 (0.42, 1.62)	0.57
	CC	0.40 (0.08, 2.09)	0.28	0.42 (0.05, 3.82)	0.44

Adjusted risk factors: gender, age, smoking, hypertension, diabetes, the ratio of LDL and HDL.

### Haplotype analysis

**Figure 2:**
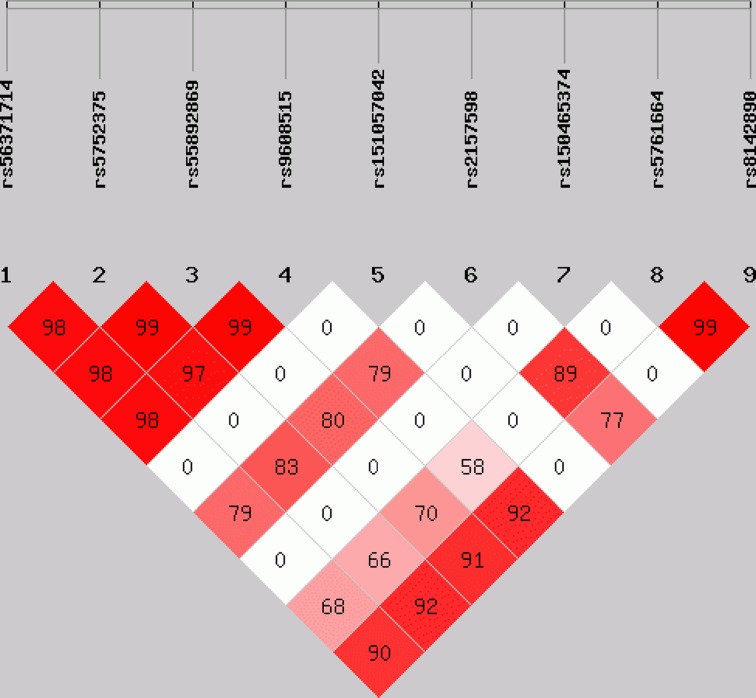
The LD tests

All polymorphisms were performed to assess LD, the results found that there was strong LD among five polymorphisms, which were rs56371714, rs5752375, rs55892869, rs9608515, rs8142890, respectively. The result of LD is shown in Figure 2. Two haplotypes were detected in five polymorphisms, no association was detected between cases and controls (Table 6).

**Table 6:** Haplotype analysis of lncRNA-MIAT polymorphisms with AMI

rs1	rs2	rs3	rs4	rs5	AMI	Controls	ORs (95% CI)	*P*-value
C	C	C	C	C	356.95 (84.2%)	379.99 (88%)	0.83 (0.54, 1.27)	0.39
T	T	T	T	T	37.96 (9%)	44.99 (10.4%)	0.87 (0.55, 1.37)	0.54

rs1, rs56371714; rs2, rs5752375; rs3, rs55892869; rs4, rs9608515; rs5, rs8142890.

### Prediction of transcription factor within the polymorphism sequence

In prediction of transcription factor, we found the polymorphisms, rs5752375 and rs9608515, in the promoter of MIAT may change the binding of transcript factor. The C base mutes T of rs5752375, results showed that the associated transcript factors FIGLA, HOXA5, NRL, PAX5, SREBF, TCF3, TFEB and USF1 may lose, however, the new transcript factors HNF4 and NKX2 may combine. In prediction of polymorphism rs9608515, the C base mutes T, the associated transcript factors EN2, GBX1, KLF5, NRF1 and THAP1 may lose, CREB1, MEIS1, NFYA, NR4A2, RORA and RORB increase.

## Discussion

Up to now, no study explored the association between polymorphisms in lncRNA-MIAT promoter with AMI, the present study provided evidence that SNPs in lncRNA-MIAT promoter correlated with AMI. Two of the ten SNPs in lncRNA-MIAT promoter (rs5752375 and rs9608515) were considered to have significantly statistical correlation with AMI in Chinese Han population in the present study. Our results revealed that the TT genotype is a risk factor for AMI compared with the CC genotype in two polymorphisms, no association between AMI with other polymorphisms rs56371714, rs55892869, rs151057042, rs2157598, rs150465374, rs5761664, rs8142890 in lncRNA-MIAT promoter was found. In addition, haplotype analysis showed no association between AMI and controls. The prediction of transcription factor within the polymorphism sequence also detected, rs5752375 and rs9608515, in the promoter of MIAT may change the binding of transcript factor.

The lncRNA-MIAT has high expression in atherosclerotic plaque [28] and low expression in ST-segment-elevation AMI patients who were treated by primary percutaneous coronary intervention than non-ST-segment-elevation MI [27]. In the animal of MI, the authors found that the level of MIAT significantly increased in peri-infract region, and they further researched that the high level of MIAT had a bad prognosis [35]. Although the mechanism of MIAT in AMI remains unclear, accumulating researches note that abnormal expression of MIAT plays a critical role in coronary atherosclerotic heart disease [27,28,36]. At present, some research has been done in molecular mechanism of lncRNA-MIAT. The competitive endogenous RNA (ceRNA) hypothesis was proposed, lncRNA/miRNA/mRNA may participate in physiological or pathological mechanism of life activity and disease [37]. Atherosclerosis generally is the major cause of AMI [38]. In an advanced atherosclerosis mouse model, Ye et al. [29] found that MIAT knockdown leads to attenuated atherosclerosis progress, reduced size of necrotic core and increased plaque stability. They performed that lncRNA-MIAT sponges miR-149-5p to inhibit efferocytosis in advanced atherosclerosis through CD47 up-regulation. Yan et al. [39] suggested that lncRNA-MIAT as a ceRNA regulates microvascular dysfunction, MIAT directly combines with miR-150-5p to regulate the vascular endothelial growth factor (VEGF). VEGF can affect cell invasion and tissue changes by inducing proteases in vascular endothelial cells [40]. After MI, the myocardial are accompanied by remodeling, including cardiac fibrosis, cardiomyocyte hypertrophy and ventricular dilation. MIAT was identified in as a risk factor, which contributes to myocardial remodeling by directly regulating miR-24 as a ceRNA to affect gene expression of Furin and TGF-β1 [35]. These results all tried to explain the key role of MIAT in regulate the expression of gene and take part in development of MI.

A few studies have explored the mechanism of MIAT expression. Ishizuka et al. [41] found some RNA-binding protein of MIAT, which may regulate the expression of MIAT. They proved that MIAT was controlled by Celf3, knockdown the Celf3 leads to the down-regulation of MIAT [41]. Although the Celf3 regulates the transcription, it is less likely that Celf3 controls the promoter sequences [41], the expression of MIAT also was controlled by promoter, the mechanism is unclear. Therefore, we try to explore the promoter sequence of MIAT, we found variants of MIAT correlated to AMI. We found rs5752375 and rs9608515 polymorphisms of MIAT promoter may as a risk factor of AMI.

The present study had a few limitations. The case–control was used and restricted the area and race, which would induce selection bias. In addition, the relatively small sample size may affect the conclusion of study, therefore, a prospective study, a large sample, diverse area and different races should be considered to verify the results. Furthermore, the conclusion needs to be confirmed by the further functional experiments.

## Conclusion

The present study presented that rs5752375 and rs9608515 polymorphisms correlated with AMI in Chinese Han population, which suggests that variants of lncRNA-MIAT gene promoter may contribute to occurrence and development of MI.

## Abbreviations

AMI: acute myocardial infarction
CAD: coronary atherosclerotic disease
ceRNA: competitive endogenous RNA
HDL: high-density lipoprotein
LD: linkage disequilibrium
LDL: low-density lipoprotein
lncRNA: long non-coding RNA
MI: myocardial infarction
MIAT: myocardial infarction association transcript
OR: odds ratio
PCR: polymerase chain reaction
SNP: single nucleotide polymorphism
TC: total cholesterol
TG: triglyceride
VEGF: vascular endothelial growth factor
95% CI: 95% confidence interval
